# Acute haemoperitoneum caused by endometriosis infiltrating the uterine artery - Two case reports and a literature review

**DOI:** 10.52054/FVVO.13.3.023

**Published:** 2021-09-24

**Authors:** A-S Vandenameele, L Platteeuw, H Alaerts

**Affiliations:** Department of Obstetrics and Gynaecology, AZ Groeninge Hospital, Kortrijk, Belgium; Department of Pathology, AZ Groeninge Hospital, Kortrijk, Belgium.

**Keywords:** endometriosis, haemoperitoneum, menstruation, uterine artery, withdrawal bleeding

## Abstract

We report 2 cases of haemoperitoneum due to a bleeding of the uterine artery caused by infiltrating endometriosis. We have also conducted a literature review on endometriosis-related intra-abdominal haemorrhage and wrote a practical guideline on how this entity can be recognized and handled.

Case 1: A 49-year-old multiparous woman presented with intense stabbing pain in the lower abdomen during her menstruation. CT angiography showed a bleeding from a side branch of the internal iliac artery. Laparoscopy was performed and an active bleeding from the right uterine artery was confirmed, clearly caused by infiltrating endometriosis lesions. Haemostasis was achieved by bipolar coagulation. Case 2: A 29-year-old nulliparous woman was admitted for observation because of heavy stabbing pain in the right lower quadrant and presence of free fluid on CT abdomen. The day after the admission, laparoscopy was performed because of a decreasing haemoglobin level. An arterial bleeding from the right parametrium was observed, probably originating from the right uterine artery. Histopathological examination of a biopsy of the right parametrium proved the presence of endometriosis. Haemostasis was achieved by bipolar coagulation.

Although endometriosis-related haemoperitoneum is a rare entity, this diagnosis should be considered when a patient presents with an intra-abdominal haemorrhage during menstruation or withdrawal bleeding - especially in case of a history or suspicion of endometriosis. Laparoscopy is the cornerstone of the treatment.

## Introduction

Endometriosis occurs in 2 to 10% of women in their reproductive years, and in 50% of women with fertility problems. It causes a chronic inflammatory reaction, which results in fibrosis and adhesions. We can differentiate three major phenotypes of lesions: superficial peritoneal gunshot lesions, endometriomas and deep infiltrating endometriosis (DIE). DIE is defined as lesions with >5mm depth of invasion (European Society of Reproduction and Embrology, 2013). They have the capacity to invade the ovaries, the uterosacral ligaments, the bladder and rectosigmoid, but blood vessels also appear to be a potential target (Dwivedi et al., 2002; Fiadjoe et al., 2008; Janicki et al., 2002). ). Haemoperitoneum caused by invasive endometriosis is a rare entity, but it is associated with important morbidity and mortality - therefore it is important to keep this diagnosis in mind. We report two cases of intra-abdominal haemorrhage due to a bleeding of the uterine artery caused by infiltration of endometriosis lesions. Both cases occurred during a period of vaginal blood loss.

## Case 1

A 49-year-old woman was brought into the emergency department because of intense stabbing pain in the lower abdomen since half an hour. She experienced already some light stabbing pain in the right iliac fossa since two days and consulted her general practitioner for this problem. Ultrasound of the abdomen the day before admission showed no abnormalities.

In the antecedents, we note treated arterial hypertension and two uncomplicated vaginal deliveries. Patient uses a triphasic oral contraceptive pill. Withdrawal bleeding started a couple of days ago.

At presentation on the emergency ward, clinical examination showed a tender abdomen, rebound tenderness and restlessness but no pain during percussion of the kidney. Pelvic examination was painful. Vital signs on admission were a blood pressure of 134/93mmHg, heart rate of 71 bpm, normal body temperature and normal saturation. Venous blood gas showed a haemoglobin level of 10.6g/dl and a mildly elevated lactate. White blood cell count was elevated (18,7*10^9/l), all other laboratory data were within normal ranges. Pregnancy test was not performed.

CT–scan visualized a large volume of free fluid but no other abnormalities. CT angiography showed an arterial blush from a side branch of the right internal iliac artery, probably concerning the gonadal vessels. The patient was admitted for observation.

**Figure 1:**
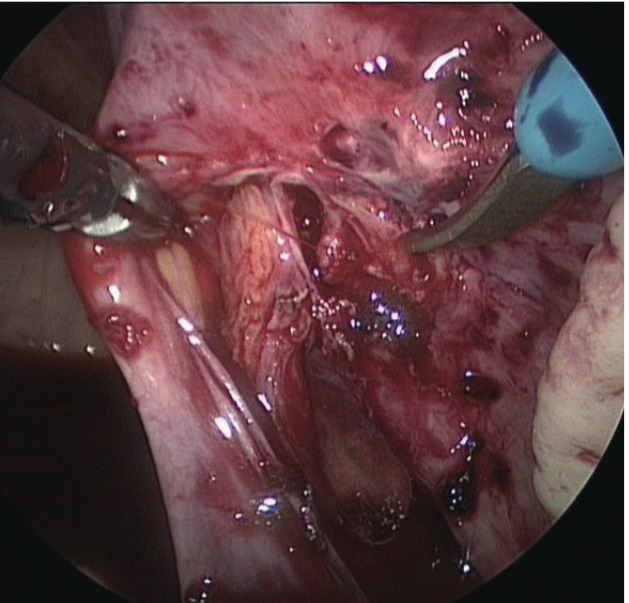
Active bleeding from the right uterine artery due to erosion of endometriosis into the artery (case 1).

**Figure 2:**
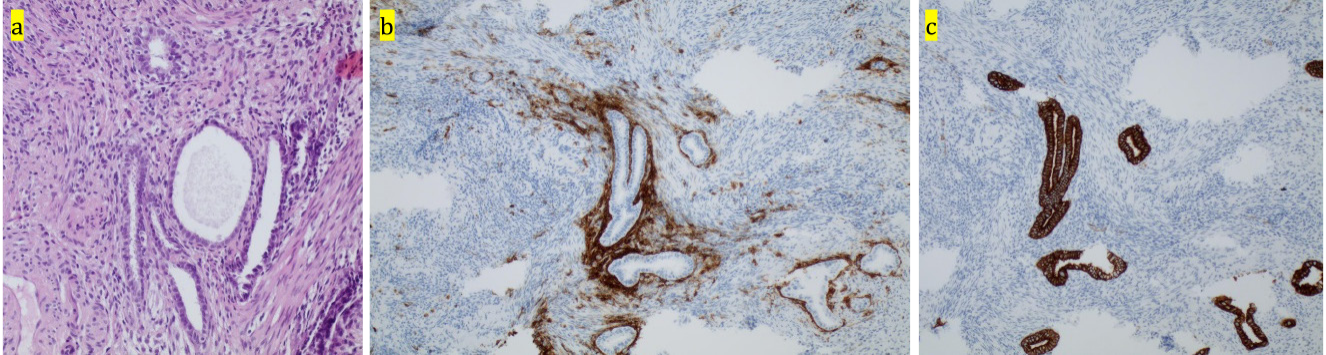
Biopsy of the deep infiltrating endometriosis nodule around the right ureter and uterine artery. (a) hematoxylin and eosin staining x 200 (b) CD10 immunohistochemistry x 200, marking the endometrial stroma (c) CK7 immunohistochemistry x 200, marking the glandular epithelium.

Haemoglobin measurement was repeated after 4 hours and had decreased to 9.4g/dl. A diagnostic laparoscopy was performed, during which we established the presence of deep infiltrating endometriosis around the right ureter with infiltration into the right uterine artery, resulting in an active bleeding of the artery (Fig.1). We also noticed superficial endometriosis lesions in the vesico- uterine pouch and in the left ovarian fossa. Figure 2 illustrates the histopathological examination of a biopsy taken from the endometriosis nodule surrounding the right ureter and uterine artery.

The arterial bleeding was stopped with bipolar coagulation after careful dissection of the right ureter. We removed 800cc of blood in total. The postoperative evolution was uneventful, haemoglobin at discharge was 9.4g/dl. We switched contraception to lynestrenol 5mg. During the postoperative follow-up in the first 6 months after surgery, the patient kept reporting pain at the right and left iliac fossa. Magnetic resonance imaging did not show any abnormalities. We performed a hysterectomy, bilateral salpingo-oophorectomy and resection of the remaining superficial endometriotic lesions in May 2020. She was pain-free after this intervention.

## Case 2

A 29-year-old woman presented herself to the emergency department in January 2020 because of intermittent intense stabbing pain in the lower right quadrant . The onset of pain was associated with heavy vaginal blood loss. The patient was taking a progestogen-only pill lynestrenol 5mg with good therapy compliance.

This patient had an extensive medical history. In 2018 she was diagnosed with endometriosis during a laparoscopy performed for a haemoperitoneum and had a laparoscopic resection of all endometriosis implants during a second procedure. In April 2019, she presented with a second haemoperitoneum, which was handled conservatively, but was complicated by an intraperitoneal infection and intestinal obstruction.

On admission, the clinical vital signs were stable with a blood pressure of 160/87mmHg, heart rate of 86 bpm, normal body temperature and normal saturation. Clinical examination showed painful deep palpation of the right iliac region and hypogastrium, but no rebound tenderness. Gynaecological pelvic examination revealed cervical motion tenderness and pain when palpating the right adnexal region.

**Figure 3:**
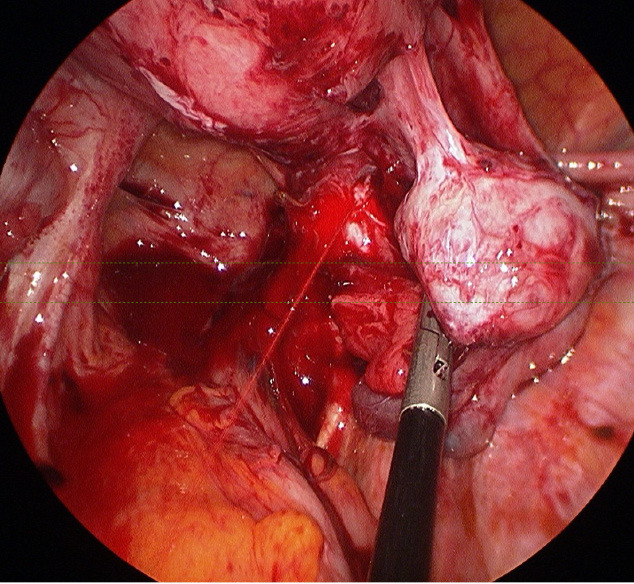
Active bleeding from the right uterine artery in the presence of multiple adhesions due to endometriosis (case 2).

**Figure 4:**
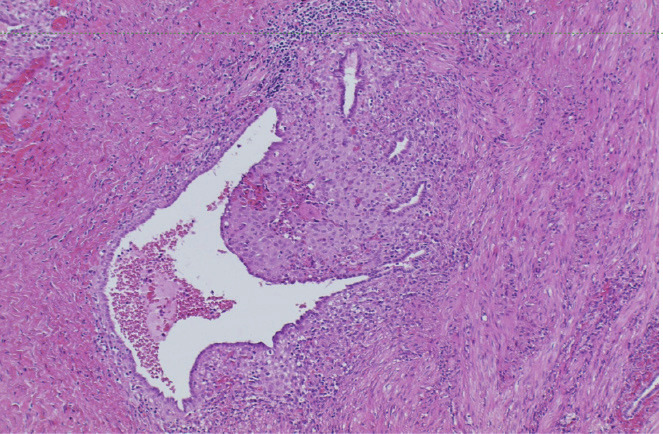
Biopsy from the right parametrium, hematoxylin and eosin staining x 100, showing endometrial glands and stroma.

Blood test results showed a haemoglobin level of 12.4g/dl, no elevation of inflammatory parameters and a negative β-HCG. A vaginal ultrasound and CT- scan were performed. Both examinations revealed free fluid in the abdomen and a homogeneous mass between the right adnexa and the uterine corpus, compatible with blood cloths. An active bleeding from the right adnexa was suspected. The patient was admitted for observation to the intensive care department. Evaluation the next day highlighted a decrease in haemoglobin level to 8.8g/dl. An exploratory laparoscopy was performed during which we observed arterial bleeding from the obliterated right ovarian fossa. After careful dissection of the adhesions between the uterus and right adnexa, we identified the source of the arterial bleeding coming from the right parametrium, at the height of the uterine artery (Figure 3). A biopsy taken from the fibrotic tissue on the right parametrium proved the presence of endometriosis (Figure 4). Haemostasis was achieved by bipolar coagulation. After identification of the right ureter, we were able to cease the arterial bleeding with bipolar coagulation. We removed a total of 900cc of blood from the abdominal cavity.

The postoperative evolution of the patient was uneventful. She received 1 unit of packed red blood cells. We continued her hormone suppression therapy of lynestrenol 5mg.

## Discussion

We searched PubMed with the terms “(endometriosis) AND (haemoperitoneum)” and checked the reference list of the retrieved articles to find similar cases of endometriosis- related acute haemoperitoneum. From the 117 hits, we selected 55 relevant publications issued between 1956 and 2020. 33 articles concerned endometriosis-associated haemoperitoneum during pregnancy, part of the known entity called “spontaneous haemoperitoneum in pregnancy” (SHiP). These publications were excluded since the pathophysiology of the bleeding in pregnant women is based on decidualisation of the endometriosis lesions and traction by uterine growth (Lier et al., 2017). Also, we have excluded 12 publications on haemorrhagic ascites as the pathophysiology of this entity is rather related to an irritation of the peritoneum than to erosive endometrial lesions. One publication was excluded because no full text was available. A summary of the 9 remaining case reports is given in Table I.

**Table I:** Case reports of haemoperitoneum caused by bleeding of endometriosis lesions outside pregnancy.

	Bleeding site	Age	Par	Therapy	Cycle	Intervention	Haemoper. (ml)
Peritoneal bleeding							
Palaia I. et al., 2020	Left utero-sacral ligament	42	NA	Dienogest	Amenorrhoea	Coagulation + haemostatic matrix	3100
Togami S. et al., 2015	Douglas peritoneum	48	≥2	No therapy	Menstruation (day 5)	Bipolar coagulation	1890
Mutihir JT et al., 2010	Posterior surface of uterus/left fallopian tube/ anterior sigmoid/Douglas	34	0	Norethisterone	Withdrawal bleeding	Coagulation + pressure packs	2000
Kumar S, 1996	Douglas/uterosacral ligaments	25	0	COCP	Amenorrhoea	Coagulation	1300
Ranney B, 1970 (11 cases: 13 cases - 2 pregn)	Ovaries/Douglas/rectosigmoid	34_avg_	4x P_0_; 7x P_≥1_	NA	Late luteal phase/ menstruation	Excision of EM and/or gyn. organs	Transfusion of 500 to 2000ml PC
Uterine/ovarian artery bleeding							
Carmichael JL et al., 1972	Arterial bleeding from the posterior surface of broad ligament	29	NA	NA	Post menstruation (day 8)	Excision of EM lesions	500
Mabrouk M et al., 2019	Left lig. ovarium proprium	35	0	No therapy	NA	Coagulation	2500
Janicki T. et al., 2002	Left uterine artery	39	0	NA	NA	Ligation	800
Fiadjoe P. et al., 2007	Right uterine artery	39	0	No therapy	Menstruation	NA	4000
Our cases							
Case 1	Right uterine artery	49	2	EE 0,03MG – gestodene	Withdrawal bleeding	Bipolar coagulation	800
Case 2	Arterial bleeding from the right parametrium	29	0	Lynestrenol	Breakthrough bleeding	Coagulation	900

Par=parity; haemoper.=haemoperitoneum; NA= information is not available; COCP= combined oral contraceptive pill; pregn=pregnancies; avg=average; EM=endometriosis; PC=packed cells; lig.=ligamentum; EE=ethinylestradiol

The molecular pathway of endometriosis is still poorly known. Gordts et al. (2017) elaborated two visions on the pathophysiology of endometriosis. The first vision stated that endometriosis is a single disease which starts as a subtle lesion that can grow, transform and undergo metaplasia over time due to repeated tissue injury and repair, caused by recurrent menstrual bleeding. The other hypothesis is that superficial lesions, endometriomas and DIE are three separate diseases, each arising from another genetic or epigenetic modified cell.

To cause bleeding from the uterine artery, the endometriotic lesions needs to infiltrate the artery wall and are by definition DIE lesions. As described by Gordts. et al. (2017), DIE lesions behave like a benign tumour; they preferably develop in the pouch of Douglas, can extend towards the uterine artery or ureters and can infiltrate the muscularis propria of different structures (Aznaurova et al., 2014; Gordts et al., 2017). The aggressive behaviour of DIE may be explained by increased proliferation activity related to oxidative stress and decreased apoptosis, by altered immunological factors and higher expression of invasive mechanisms (f. ex. expression of matrix metalloproteinases, nerve growth factor, vascular endothelial growth factor and intercellular adhesion molecule) (Rolla et al., 2019; Tosti et al., 2015).

When establishing the presence of free fluid, it is important to identify whether it concerns blood or ascites fluid. Repeated haemoglobin measurements, vaginal ultrasound, CT-scan or optionally a culdocentesis can help to make this distinction. This enables us to make a first step in the differential diagnosis - ascites originates from carcinomatous peritonitis, liver cirrhosis, portal vein thrombosis or heart failure (El Khalil et al. 1999); free blood can be the consequence of a ruptured ectopic pregnancy, ruptured ovarian cysts i.a. endometriomas, a corpus luteum or other non-gynaecological pathology.

Furthermore, the timing of haemoperitoneum onset during the menstrual cycle can be a clue in the differential diagnosis. Endometriosis is an oestrogen- dependent disease as oestrogens stimulate the growth of the lesions. The trigger for bleeding of the implants is most likely a state of progesterone withdrawal, i.e. menstruation or a withdrawal bleeding induced by cessation of progestogen-only medication (POP) or a combined contraceptive. In both of our cases the haemorrhage also occurred during a period of vaginal blood loss. However, in our second case, it was associated with breakthrough bleeding while the patient was taking lynestrenol with good therapy compliance. This patient already switched her hormone- suppressive therapy five times because of recurrent menometrorrhagia. Palaia et al. (2020) described a similar case of endometriosis-related haemoperitoneum during breakthrough bleeding – one of their hypotheses was that it was related to the development of a resistance to progestins. Reis et al. (2020) investigated why progestins have a variable therapeutic response in patients with endometriosis. Part of the answer is that the progesterone receptor (PR) expression appears to be reduced and disrupted in endometriotic foci, due to congenital or epigenetic abnormalities affecting the PR gene transcription and oxidative stress (Reis et al., 2020).

The haemorrhage most commonly originates from endometriosis lesions at the posterior surface of the uterus and on the utero-ovarian vessels in the parametrium, but we have to note that in many cases the exact source of bleeding can not be identified (Lier et al., 2017). The bleeding is not always the result of erosive lesions, it can also be the result of fibrosis due to endometriosis or previous interventions.

At presentation, vital signs should be checked immediately and any sign of hypovolemic shock should be recognised. The patient typically presents with pelvic pain, sometimes preceded by episodes of stabbing pain in the lower abdomen in the days before presentation. The pain increases progressively and spreads quickly over the entire abdomen. Peritoneal irritation is often present during clinical examination. Blood test show a decreased level of haemoglobin and can be repeated after a couple of hours to evaluate active blood loss. As an ectopic pregnancy is the most common cause of haemoperitoneum in women of childbearing age, a pregnancy test should always be performed. No test was conducted in our first case, which can be considered a medical error.

Vaginal ultrasound is an easy first-line examination to identify the presence of free fluid and to occasionally identify the origin of the bleeding. This examination can be repeated after a few hours to determine whether the amount of free fluid has increased. CT-scan should be performed to rule out other causes of haemoperitoneum and to indicate the site of bleeding if possible. CT angiography can be of additional value to identify the exact origin of active bleeding and to evaluate the possibility of embolisation. In our first case, the patient was immediately assigned to the emergency physicians as she had come in by ambulance. Given the severity of her symptoms, a CT scan was performed immediately. The scan showed an ample amount of free fluid, so an additional vaginal ultrasound did not seem useful. The second patient was immediately allocated to a gynaecologist, who performed a vaginal ultrasound that established the presence of free fluid. An additional CT-scan was performed to further clarify the situation.

Depending on the haemodynamic condition of the patient, an expectant management can be adopted with repeated haemoglobin measurement and ultrasound after a couple of hours. In most cases a surgical approach is needed. First choice is a laparoscopy with use of coagulation, clipping and/or a haemostatic matrix to stop the bleeding. When the patient deteriorates quickly or when the bleeding site can not be identified during laparoscopy, the operation needs to be converted to a laparotomy.

**How to recognize and approach an endometriosis-related haemoperitoneum?**

- (sub)acute abdomen during menstruation, withdrawal bleeding or breakthrough bleeding
- History or suspicion of endometriosis
- Presence of free fluid – make a distinction between blood and ascites
- Negative pregnancy test
- Ultrasound/CT (angiography) to identify the origin of bleeding
- Expectant management vs. laparoscopy with bipolar coagulation

## Conclusion

Haemoperitoneum caused by infiltrating endometriosis is a rare entity, but the diagnosis should be given consideration when a patient presents with an intra-abdominal haemorrhage during a period of vaginal blood loss - after ruling out an ectopic pregnancy, a bleeding from a corpus luteum or ruptured ovarian cyst and other obvious non-gynaecological pathology. The bleeding can originate from peritoneal implants, but can also emerge from infiltrating lesions into the utero-ovarian vessels. In the latter situation, the patient can deteriorate quickly. In most cases, a laparoscopy needs to be performed to achieve haemostasis.
